# Exploring the Sorption Mechanism of Ni(II) on Illite: Batch Sorption, Modelling, EXAFS and Extraction Investigations

**DOI:** 10.1038/s41598-017-09188-z

**Published:** 2017-08-17

**Authors:** Xiaolan Zhao, Shirong Qiang, Hanyu Wu, Yunbo Yang, Dadong Shao, Linchuan Fang, Jianjun Liang, Ping Li, Qiaohui Fan

**Affiliations:** 1 Key Laboratory of Petroleum Resources Research, Institute of Geology and Geophysics, Chinese Academy of Sciences, Lanzhou, Gansu Region 730000 China; 2 Key Laboratory of Preclinical Study for New Drugs of Gansu Province, and Institute of Physiology, School of Basic Medical Sciences, Lanzhou University, 199 Donggang West Road, Lanzhou, 73000 China; 3 Institute of Plasma Physics, Chinese Academy of Sciences, Hefei, 230031 China; 4 State Key Laboratory of Soil Erosion and Dryland Farming on the Loess Plateau, Northwest A&F University, Yangling, 712100 China; 5 University of Chinese Academy of Sciences, Beijing, 100049 China

## Abstract

The sorption mechanism of nickel (Ni) at the illite/water interface was investigated using batch, sorption modelling, extended X-ray absorption fine structure (EXAFS), and extraction approaches. The results showed that Ni(II) sorption on illite was strongly dependent on pH, contact time, temperature, and initial Ni(II) concentration. At a low initial Ni(II) concentration, the ion exchange species of ≡X_2_Ni° and the inner-sphere complexes including ≡S^s^ONi^+^, ≡S^w^ONi^+^ and ≡S^w^ONiOH° species are observed on the sorption edges of Ni(II) on illite. As the initial Ni(II) concentration increased to 1.7 × 10^−3^ mol/L, precipitates including surface-induced precipitation of s-Ni(OH)_2_ and amorphous Ni(OH)_2_ became more significant, especially under neutral to alkaline conditions. EXAFS analysis confirmed that Ni-Al layered double hydroxide (LDH) can gradually form with an increase in the contact time. At pH 7.0, α-Ni(OH)_2_ was produced in the initial stage and then transformed to the more stable form of Ni-Al LDH with increasing contact time because of the increased Al^3+^ dissolution. With an increase in temperatures, α-Ni(OH)_2_ phase on illite transformed to Ni-Al LDH phase, indicating a lower thermodynamic stability compared to Ni-Al LDH phase. These results are important to understand the geochemical behaviors to effectively remediate soil contaminated with Ni(II).

## Introduction

Nickel (Ni) is one of the most toxic and widespread contaminants in the environment. There have been vast quantities of nickel released into the soil and water from industrial production such as mining, oil refining, electroplating, and battery and accumulator manufacturing^1, 2^. Once released into the environment, nickel readily transports and migrates as a soluble form of Ni^2+^ or Ni(II)-ligand complexes, which are very important and directly related to Ni(II) toxicity in environmental media. Therefore, Ni(II) can be easily assimilated by terrestrial and aquatic organisms and can gradually accumulate in the biological food chain. Although trace nickel is one of the essential elements for many organisms, a high level of Ni(II) is toxic and can cause cancer, nausea, vomiting, diarrhea, skin dermatitis, renal edema, and pulmonary fibrosis^3–5^. Therefore, the study of Ni(II) speciation and bioavailability at a solid/water interface is important for the prediction and evaluation of its ecological and environmental risks and toxicity and to the remediation of Ni(II)-contaminated soil and water.

Generally, the migration and retention of Ni(II) in contaminated soil and groundwater is largely controlled by its sorption and desorption behaviors at the solid/water interface. Previous studies have demonstrated that Ni(II) strongly and selectively interacted with phyllosilicate minerals that were widely distributed in the soil and sediments, such as montmorillonite^6–15^, kaolinite^16, 17^, bentonite^18, 19^, attapulgite^1, 20^, diatomite^21^, and illite^2, 22, 23^. These studies showed that environmental factors such as pH, ionic strength, and temperature can affect the sorption/desorption behaviors of Ni(II) to a large extent at both the micro- and macro-scales. Moreover, ion exchange (IE) or the outer-sphere complexes (OSCs) were the primary mechanisms of Ni(II) sorption on clay minerals at a low pH, whereas the inner-sphere complexes (ISCs), especially surface precipitates, were possibly dominant mechanisms for Ni(II) retention in the neutral to high pH range. Some studies found various surface precipitates, such as Ni(II) hydroxide, Ni-Al layered double hydroxides (LDHs) and Ni-phyllosilicate, under neutral to weak alkaline conditions^21, 24–34^. The formation of these mixed hydroxide phases can significantly stabilize Ni(II) in environmental media and notably decrease the Ni(II) mobility and bioavailability^31^. Therefore, it is crucial to understand the nature of these surface precipitates because their solubility depends on their structure and composition.

Extended X-ray absorption fine structure (EXAFS) was confirmed to be useful in discerning and providing important insights to these different surface species and precipitates by investigating the different features of Ni-O and the Ni-Ni/Al/Si distance (*R*) and coordination number (*CN*). Previous studies have reported that Ni-phyllosilicate (co)precipitates on solid surfaces with a Ni-Ni interatomic distance of 3.07 to 3.10 Å and a Ni-Si interatomic distance of ~3.28 Å^21^, α-Ni(OH)_2_ with a Ni-Ni interatomic distance of 3.07 to 3.09 Å^27, 35^, and β-Ni(OH)_2_ with a Ni-Ni interatomic distance of 3.11–3.13 Å^21, 25, 27, 32, 33^. The Ni-Al LDH is quite stable and insoluble, resulting in a reduction of the Ni(II) bioavailability in the environment^31^, where Ni is surrounded by several Ni atoms at distances ranging from 3.05 Å to 3.08 Å and Al atoms at distances ranging from 3.03 Å to 3.12 Å^27, 30^. However, whether Ni-Al LDH can form on a clay surface strongly depends on the availability of Al^3+^ during the dissolution of Al-containing sorbents. Previous studies have confirmed that Ni-Al LDH can form very quickly within several minutes, which is questionable given that mineral dissolution is generally slow.

Illite, which is one of the most dominant clay minerals in soil and sediments, forms from silicate weathering through the alteration of other clay minerals and the degradation of muscovite. The structure is a 2:1 clay with silica tetrahedron-alumina octahedron-silica tetrahedron layers and has a large specific surface area. Its high sorption capacity for cations via ion exchange and surface complexation has a major impact on metal ion retention in the environment. To the best of our knowledge, discussions of the relationship between Ni(II) speciation and availability at the illite/water interface is scarce, especially using the EXAFS technique; however, this relationship is critical to understand the geochemical behaviors of Ni(II) in the environment. In addition, the primary focus of previous studies on Ni sorption at low concentrations is unsuitable for significantly contaminated soils close to the metal ores. Therefore, the sorption mechanism and speciation of Ni(II) at the illite/water interface will be explored by batch, sorption modelling and EXAFS approaches in this work. The results of the relationship between species and availability in this study will provide important insights into the understanding and prediction of Ni(II) geochemical behaviors in soils enriched in phyllosilicate clays such as illite.

## Results and Discussion

### Ni(II) sorption on illite

**Figure 1:**
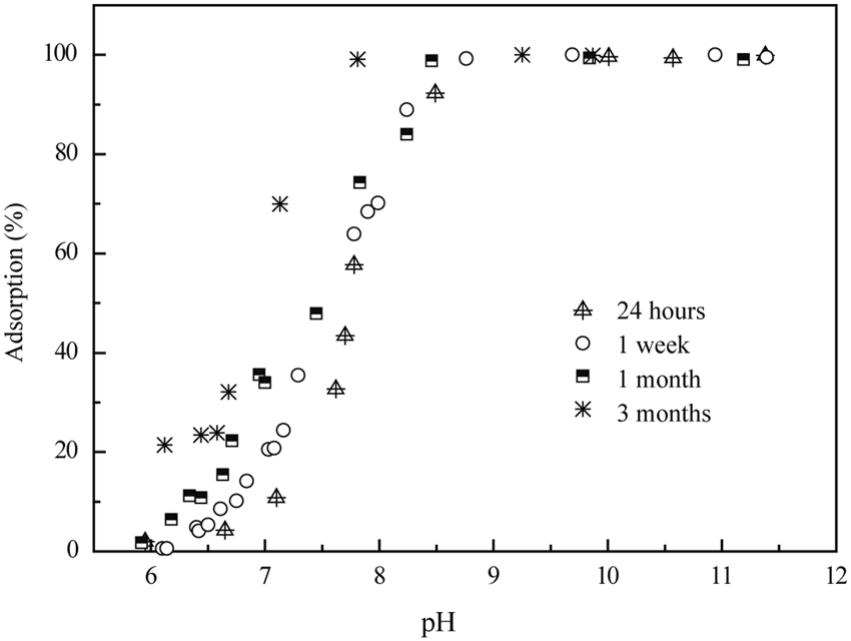
Sorption of Ni(II) on illite as a function of contact time. *C*_Ni(II)initial_ = 1.7 × 10^−3^ mol/L, Solid to liquid ratio (*S/L*) = 2.0 g/L, *I* = 0.1 M NaClO_4_, *T* = 298 K.

The uptake of Ni(II) on illite as a function of pH was examined at different contact times of 24 hours, 1 week, 1 month and 3 months (Fig. 1). The sorption percentage of Ni(II) increased as the contact time increased from 24 hours to 3 months. Fan *et al*.^1^, Hu *et al*.^2^, and Sheng *et al*.^21^ showed that the initial sorption of Ni(II) was very quick on attapulgite (2 h), illite (5 h) and diatomite (4 h) and continued at much slower sorption rates, demonstrating that strong surface complexation or (co)precipitates contributed to the Ni(II) sorption on illite.

**Figure 2:**
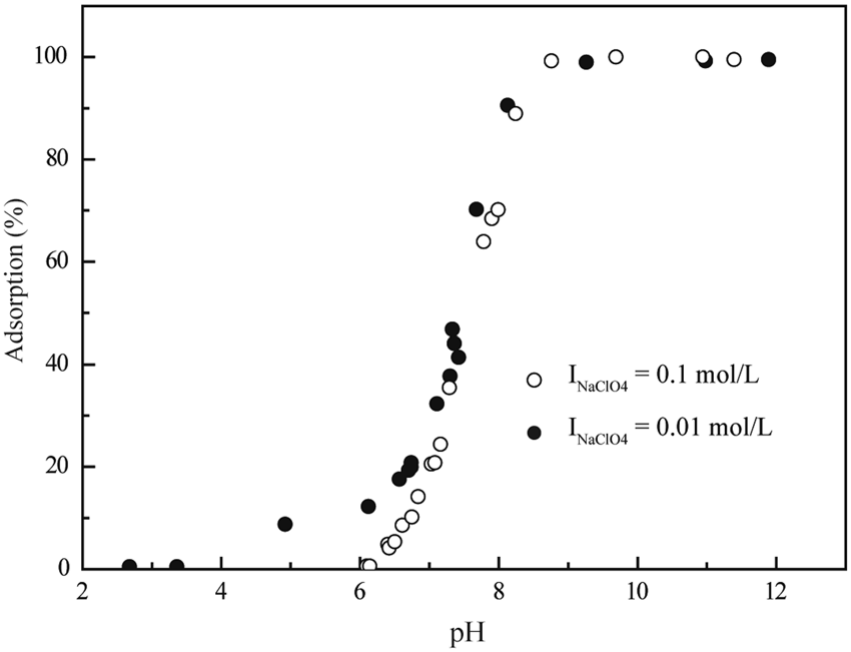
Effect of ionic strength on Ni(II) sorption to illite as a function of pH values. *C*_Ni(II)initial_ = 1.7 × 10^−3^ mol/L, *S/L* = 2.0 g/L, *I* = 0.1 M NaClO_4_, *T* = 298 K.

Figure 1 shows that Ni(II) sorption on illite was clearly affected by the pH values, which is a very important factor in the environment. From pH 6.0 to 7.0, the sorption of Ni(II) on illite was lower and ranged from 1.0% to 10.0%. However, over 90% of the Ni(II) was adsorbed on illite above pH 8.0. The strong pH-dependence suggested that both OSCs and ISCs possibly control Ni(II) sorption behaviors on illite. Echeverria *et al*.^22^ and Hu *et al*.^2^ concluded that the ISCs were the primary mechanism under neutral to weak alkaline conditions. Figure 2 shows the Ni(II) sorption percentage on illite as a function of pH in 0.01 M and 0.1 M NaClO_4_ solutions. Ni(II) sorption decreased with the increase in ionic strength below pH 8.0. This suggested that IE or OSCs were dominant mechanisms in the low pH range because both are very sensitive to the ionic strength^36^. No significant difference was observed at different ionic strength conditions above pH 8.0.

According to the solubility product of Ni(OH)_2_ (2.0 × 10^−15^)^2^, one can deduce that the formation of Ni(OH)_2_ can occur only above pH 8.0 at the concentration of 1.7 × 10^−3^ mol/L if no Ni(II) is adsorbed on the illite surface. This indicated that, in theory, the formation of Ni(OH)_2_ did not contribute to the sorption of Ni(II) on illite below pH 8.0. However, recent studies have confirmed the presence of Ni(OH)_2_ and Ni-Al or Si LDH on clay and oxide surfaces^21, 24–34^. For example, Scheidegger and Sparks^25^ observed the formation of mixed-cation hydroxide phases produced by metal sorption on clays and aluminum oxides. These hydroxide surface phases formed far below the theoretical monolayer coverage and in a pH range well below the pH where metal hydroxide precipitates would be expected according to the thermodynamic solubility product. Therefore, another possibility for the Ni(II) sorption mechanisms on illite, i.e., surface-induced precipitation, cannot be excluded given the limited sorption data, which will be discussed in detail later by combining the sorption modelling and spectroscopies analyses.

### Sorption modelling of Ni(II) on illite

**Figure 3:**
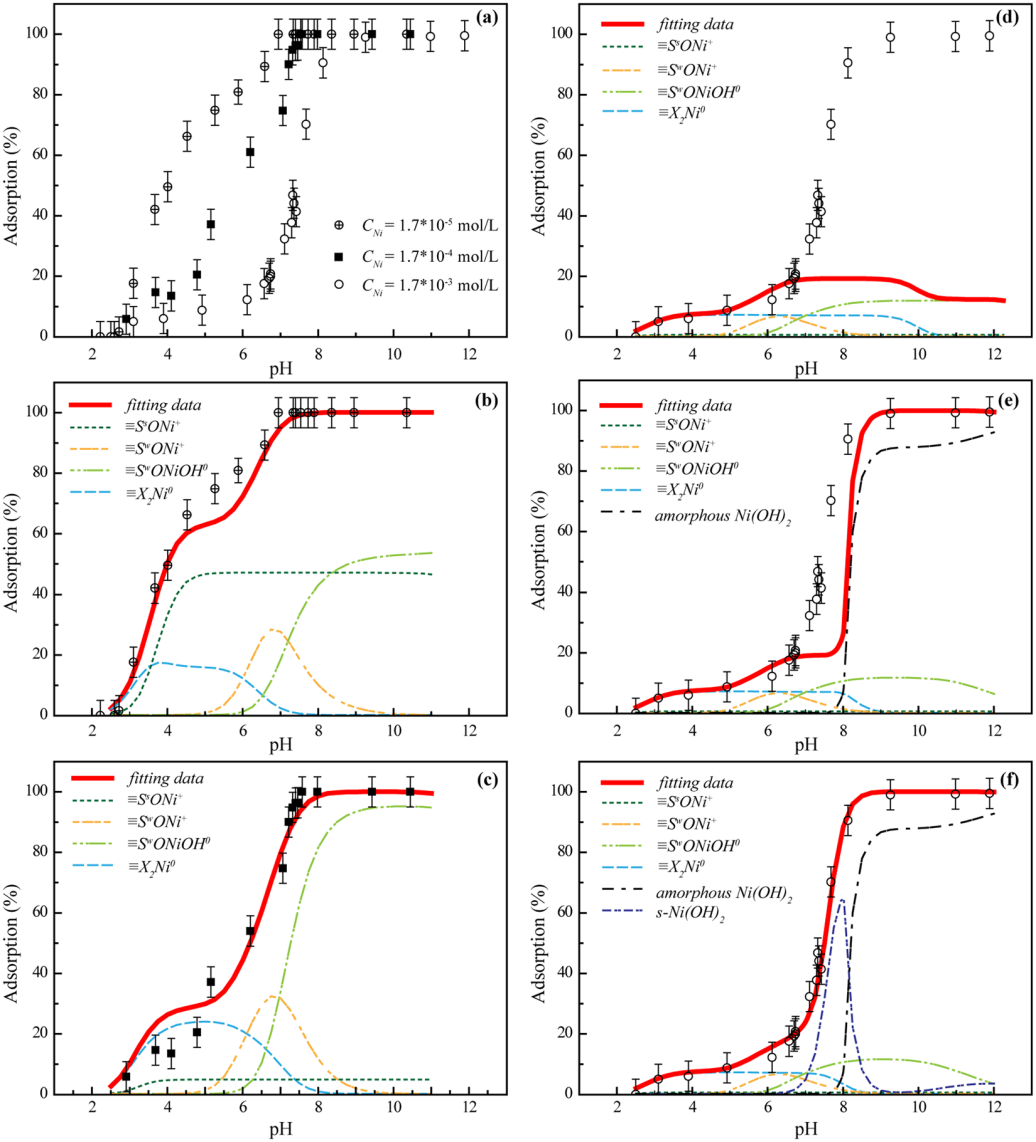
Sorption and modelling of Ni(II) on illite as a function of pH at different initial Ni(II) concentrations. (**a**) Sorption edges on illite at different initial Ni(II) concentrations. (**b**) Sorption model at *C*_Ni(II)initial_ = 1.7 × 10^−5^ mol/L. (**c**) Sorption model at *C*_Ni(II)initial_ = 1.7 × 10^−4^ mol/L. (**d**–**f**) Sorption model of cases 1, 2, and 3 at *C*_Ni(II)initial_ = 1.7 × 10^−3^ mol/L, *S/L* = 2.0 g/L, *I* = 0.01 M NaClO_4_. All samples were tightly capped in centrifuge tubes and shaken for 1 week.

The sorption edges of Ni(II) on illite were estimated in a wide range of initial Ni(II) concentrations from 1.7 × 10^−5^ to 1.7 × 10^−3^ mol/L (Fig. 3). Figure 3a shows that the sorption edge of Ni(II) on illite was significantly shifted forward to a high pH range (approximately 2.0 pH units) as the initial Ni(II) concentration increased from 1.7 × 10^−5^ to 1.7 × 10^−3^ mol/L. To explore the sorption species and mechanism of Ni(II) on illite, the surface complexation model and MINTEQ 3.1 code were combined to study the sorption edges at the different initial Ni(II) concentrations, and the fit results are shown in Fig. 3 and Table 1. For the sorption system with the initial Ni(II) concentration of 1.7 × 10^−5^ mol/L (Fig. 3b), three surface species including ≡X_2_Ni°, ≡S^s^ONi^+^, and ≡S^w^ONi^+^ were dominant below pH ~6.5, and ≡S^w^ONiOH° was primarily responsible for the sorption above pH ~7.0. The results showed that IE was important to Ni(II) sorption on illite at a low pH range and that ISCs primarily controlled Ni(II) sorption over a high pH range. The relative sorption reaction constants were comparable to the previous studies^37–39^, which indicated that the sorption model was reasonable. To further validate the sorption model under different conditions, the model and the corrected parameters were directly extrapolated to the systems with higher initial Ni(II) concentrations. The fitting results showed that the sorption model can reasonably predict the sorption edge of Ni(II) on illite at *C*_Ni_ = 1.7 × 10^−4^ mol/L, although the contributions of each surface species were changed in comparison with the sorption system at *C*_Ni_ = 1.7 × 10^−5^ mol/L.

**Table 1:** Sorption Model of Ni(II) to Illite Using the MINTEQ 3.1 Code.

Site Type	Site Capacities (mol/kg)
≡S^s^OH	4.0 × 10^−3^
≡S^w^OH	1.0 × 10^−1^
Cation exchange capacity	2.0 × 10^−1^
Surface ion exchange and acid-base reactions	log K
≡S^s^OH ↔ ≡S^s^O^−^ + H^+^	−9.5
≡S^s^OH + H^+^ ↔ ≡S^s^OH_2_^+^	6.3
≡S^w^OH ↔ ≡S^w^O^−^ + H^+^	−8.3
≡S^w^OH + H^+^ ↔ ≡S^w^OH_2_^+^	5.1
≡XH + Na^+^ ↔ ≡XNa + H^+^	1.0
Surface complexation reactions	log *K*
≡S^s^OH + Ni^2+^ ↔ ≡S^s^ONi^+^ + H^+^	2.8
≡S^w^OH + Ni^2+^ ↔ ≡S^w^ONi^+^ + H^+^	−2.3
≡S^w^OH + Ni^2+^ + H_2_O ↔ ≡S^w^ONiOH^0^ + 2 H^+^	−9.5
*s*-Ni^2+^ + 2H_2_O = *s*-Ni(OH)_2_ + 2 H^+^	−15.0
Ni^2+^ + 2H_2_O = Ni(OH)_2_ + 2 H^+^	−14.7
Cation exchange reaction	log *K*
≡2XNa + Ni^2+^ ↔ ≡X_2_Ni^0^ + 2Na^+^	0.5

However, the above sorption model cannot predict Ni(II) sorption behaviors at *C*_Ni_ = 1.7 × 10^−3^ mol/L. Conversely, the total capacities of the sorption sites and cation exchange site were limited and far lower than the Ni(II) concentration in the illite phase at the highest Ni(II) concentration (i.e., *C*_Ni_ = 1.7 × 10^−3^ mol/L); thus, it is necessary to consider other sorption mechanisms in this model. In this study, three cases were considered to tentatively describe the sorption edge of Ni(II) on illite as a function of pH for the system with the highest Ni(II) concentration (*C*_Ni_ = 1.7 × 10^−3^ mol/L, Fig. 3d–f). Case 1: only IE and surface complexation; case 2: IE, surface complexation, and amorphous Ni(OH)_2_; and case 3: IE, surface complexation, amorphous Ni(OH)_2_ and surface-induced precipitation of s-Ni(OH)_2_ were analyzed for their contributions to Ni(II) sorption over the entire observed pH range. Figure 3d shows that all sorption sites and IE were saturated when the sorption was approximately 15% at pH ~7.0. However, the primary species of ≡X_2_Ni°, ≡S^w^ONi^+^ and ≡S^w^ONiOH° can explain the sorption trend below pH 6.5. The strong site contribution is negligible for Ni(II) sorption, due to its limited site capacity (approximately 4.0% that of the weak site, see Table 1). Above pH 6.5, the fit data deviated far from the experimental data, indicating that some important sorption species were missed for case 1. As discussed in the sorption process, amorphous Ni(OH)_2_ possibly played a very important role in the sorption of Ni(II), especially in the high Ni(II) concentration and pH ranges. Therefore, in case 2, the amorphous precipitate Ni(OH)_2_ was included in the sorption model, and the fit data were plotted (Fig. 3e). Calculations show that the amorphous Ni(OH)_2_ began to form above pH 8.0. Case 2, which included the amorphous Ni(OH)_2_ contribution to Ni(II) sorption on illite had improved results compared to case 1; however, there were still fitting vacancies in the pH range from 6.5 to 8.0.

Attempting to improve the model in this region, one type of surface-induced precipitate (s-Ni(OH)_2_) was considered in case 3. These surface-induced precipitates, which were reported in previous studies and observed in the EXAFS spectra in this study, were formed and were possibly dominant for Ni(II) retention in the neutral to high pH range^21, 24–34^. Figure 3f shows that five different species were necessary to describe the sorption edge of Ni(II) on illite at *C*_Ni_ = 1.7 × 10^−3^ mol/L. Below pH 6.5, the two species ≡X_2_Ni° and ≡S^w^ONi^+^ were predominant, and the sorption were ~30% and 70%, respectively. In the pH range from 7.0 to 9.0, s-Ni(OH)_2_, amorphous Ni(OH)_2_ and the ISCs of ≡S^w^ONiOH° were responsible for Ni(II) sorption. Above pH 9.0, the ISCs of ≡S^w^ONiOH° and amorphous Ni(OH)_2_ were predominant. The sorption species and precipitates of Ni(II) at the illite/water interface were confirmed in the observed pH range by X-photoelectron spectroscopy in the supplemental information (SI).

### EXAFS analysis of reference samples

**Figure 4:**
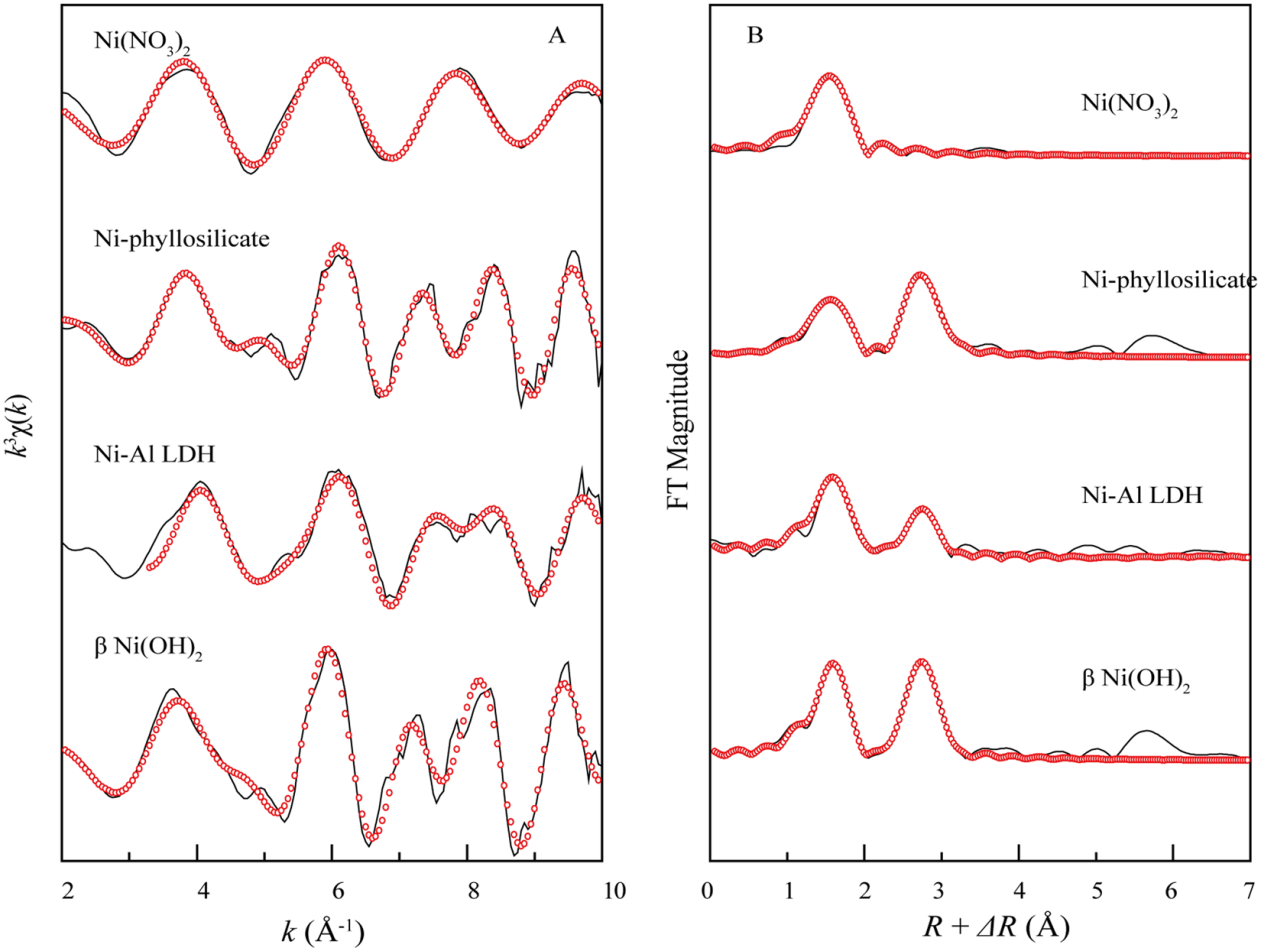
EXAFS spectra of standard samples. (**A**) *k* ^3^χ(*k*) functions and (**B**) corresponding RSFs (phase shift uncorrected). Solid line: experimental data; red dotted line: fitted data.

EXAFS spectra and the radial structure functions (RSFs) of reference samples Ni(NO_3_)_2_, Ni-phyllosilicate, Ni-Al LDH, and β-Ni(OH)_2_ are shown in Fig. 4. Figure 4A shows a monotonous feature of Ni(NO_3_)_2_ in the *k* ^3^χ(*k*) function at k > 3.0 Å^−1^, indicating that Ni(NO_3_)_2_ possessed a single coordination environment surrounding the Ni atoms that was primarily the hydration shell. The *k* ^3^χ(*k*) functions of Ni-phyllosilicate, Ni-Al LDH and β-Ni(OH)_2_ have more complicated oscillation features. At 5.0 Å^−1^, there was a small beat in the *k* ^3^χ(*k*) function; notably, there was a typical split in the *k*-range of ~7.5 Å^−1^, which is characteristic of Ni precipitation and is due to the neighboring atoms. Therefore, the sorption speciation was accurately distinguished from these characteristic oscillations in the *k* ^3^χ(*k*) functions. Figure 4B shows the corresponding RSFs of the reference samples. For Ni(NO_3_)_2_, only one coordinated shell at approximately 2.0 Å (phase shift uncorrected) was observed in the RSFs assigned to the Ni-O shell, which was the hydration shell. For the samples of Ni-phyllosilicate, Ni-Al LDH and β-Ni(OH)_2_, the second coordinated shells were observed at approximately 3.08 Å and were assigned to the Ni-Ni, Ni-Si or Ni-Al scattering pairs^25, 30, 33, 34^. As shown in the RSFs (Fig. 4B), the intensities of the second shells were different for each Ni precipitate, which was reflected in the *k* ^3^χ(*k*) functions and could be an important criterion to identify the type of precipitates.

The structural parameters of the reference samples are specified in Table 2. Ni(NO_3_)_2_ was surrounded by 6.0 O atoms at *R*_Ni-O_ ≈ 2.04 Å. For the complex features of backscattering, Ni-phyllosilicate was surrounded by 4.5 O atoms at *R*_Ni-O_ ≈ 2.03 Å in the first shell and by 2.7 Ni atoms at *R*_Ni-Ni_ ≈ 3.07 Å and 4.0 Si atoms at *R*_Ni-Si_ ≈ 3.27 Å. For Ni-Al LDH, Ni was surrounded by 6.0 O atoms at *R*_Ni-O_ ≈ 2.03 Å in the first shell and by 3.5 Ni atoms at *R*_Ni-Ni_ ≈ 3.06 Å and 2.5 Al atoms at *R*_Ni-Al_ ≈ 3.12 Å. For Ni(OH)_2_, Ni was surrounded by 6.0 O atoms at *R*_Ni-O_ ≈ 2.05 Å and by 6.0 Ni atoms at *R*_Ni-Ni_ ≈ 3.11 Å. The reference samples can be simulated well by combining the *CN* and *R* parameters, which are well correlated with previous studies^25, 30, 33, 34^.

**Table 2:** Structural Parameters Derived from EXAFS Analysis for the Reference and Sorption Samples.

Sample conditions	First shell (Ni-O)			Second shell (Ni-Ni/Al/Si)			
	CN	R(Å)	σ^2^(Å^2^)		CN	R(Å)	σ^2^(Å^2^)
(a) Ni(NO_3_)_2_	6.0	2.04	0.007				
(b) Ni-phyllosilicate	4.5	2.03	0.009	Ni-Ni	2.7	3.07	0.005
				Ni-Si	4.0	3.27	0.007
(c) Ni-Al LDH	6.0	2.03	0.008	Ni-Ni	3.5	3.06	0.005
				Ni-Al	2.5	3.12	0.003
(d) β-Ni(OH)_2_	6.0	2.05	0.006	Ni-Ni	6.0	3.11	0.008
(e) pH 6.0, 298 K, 1 day	6.0	2.06	0.007	Ni-Si	3.3	3.23	0.003
(f) pH 6.0, 298 K, 1 month	6.4	2.06	0.006	Ni-Ni	5.2	3.08	0.01
				Ni-Al	0.8	3.14	0.008
(g) pH 7.0, 298 K, 1 day	5.9	2.05	0.008	Ni-Ni	4.0	3.07	0.009
(h) pH 7.0, 298 K, 1 month	6.0	2.06	0.004	Ni-Ni	5.2	3.07	0.006
				Ni-Al	1.8	3.14	0.009
(i) pH 10.0, 298 K, 1 day	6.0	2.06	0.006	Ni-Ni	6.0	3.13	0.007
(j) pH 10.0, 298 K, 1 month	6.0	2.06	0.004	Ni-Ni	6.0	3.13	0.007
(k) pH 7.0, 303 K, 1 day	6.1	2.06	0.005	Ni-Ni	5.6	3.08	0.008
				Ni-Al	1.5	3.14	0.008
(l) pH 7.0, 313 K, 1 day	5.7	2.06	0.004	Ni-Ni	5.7	3.08	0.006
				Ni-Al	3.2	3.14	0.007

*CN*, coordination number; *R*, interatomic distance; *σ* ^2^, Debye-Waller factor.

### EXAFS analysis on the effects of contact time and pH

**Figure 5:**
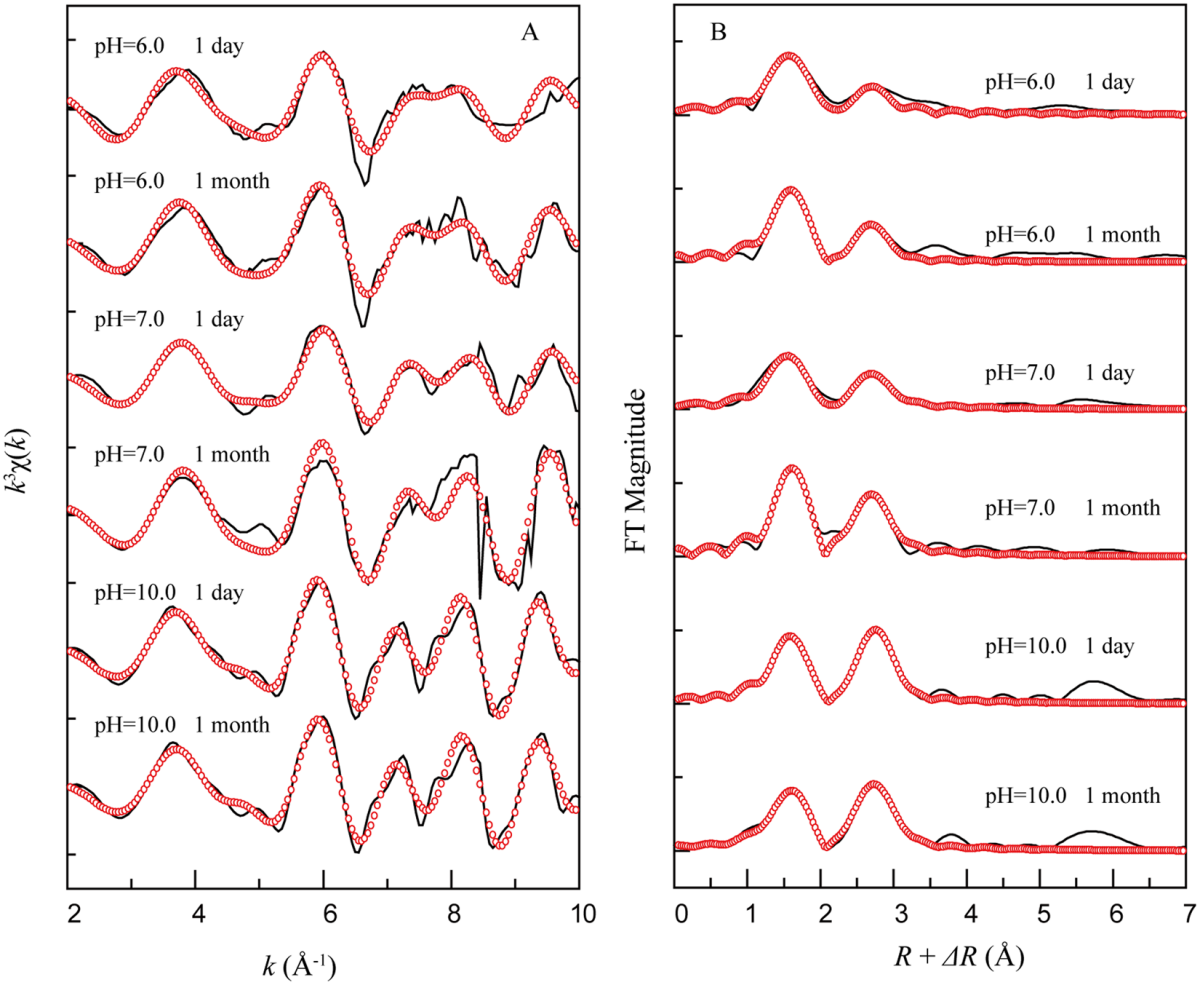
Nickel K-edge EXAFS spectra for Ni(II) adsorbed illite under different pH values. (**A**) *k* ^3^χ(*k*) functions and (**B**) corresponding RSFs (phase shift uncorrected). Solid line: experimental data; red dotted line: fitted data. *C*_Ni(II)initial_ = 1.7 × 10^−3^ mol/L, *pH* = 6.0, 7.0, 10.0, *I* = 0.01 M NaClO_4_, *S/L* = 0.16 g/L, *T* = 298 K.

Figures 5A and 5B show the *k* ^3^ *χ*(*k*) functions and the corresponding RSFs of the sorption samples for different contact times and pH values. A complicated oscillation feature was observed, especially at k ≥ 5.0 Å^−1^, indicating the presence of higher coordinated shells surrounding the Ni atoms, with an exception of the Ni-O shell. At different contact times, the *k* ^3^ *χ*(*k*) functions were characterized by multi-frequency wave shapes with distinct features (Fig. 5A). The splitting degree was enhanced with an increasing contact time from 1 day to 1 month at pH 6.0 and 7.0; however, no clear change was observed for the Ni(II)-adsorbed illite at pH 10.0. Moreover, the oscillations for the samples at pH 10.0 were extremely similar to the β-Ni(OH)_2_ reference and were independent of the contact time, which suggested that β-Ni(OH)_2_ was the dominant sorption mechanism for Ni(II) sorption on illite under strong alkaline conditions.

As expected, two typical coordinated shells in the *R* space were observed for all the samples at different pH values and contact times (Fig. 5B). The second shell for the samples at pH 6.0 and 7.0 increased as the contact time increased, which indicated that the sorption mechanism possibly changed completely from the IE and/or ISCs at pH 6.0 and α-Ni(OH)_2_ at pH 7.0 to surface precipitation such as Ni-Al LDH as the contact time increased. No changes in the RSFs for the Ni(II)-adsorbed illite at pH 10.0 suggested that the primary sorption mechanism did not change. This is consistent with the XPS analysis, showing that IE and ISCs are dominant in a low pH range and that the precipitates, including Ni(OH)_2_ and Ni-Al LDH, are contributing to Ni(II) sorption under alkaline conditions (Fig. SI-6).

**Figure 6:**
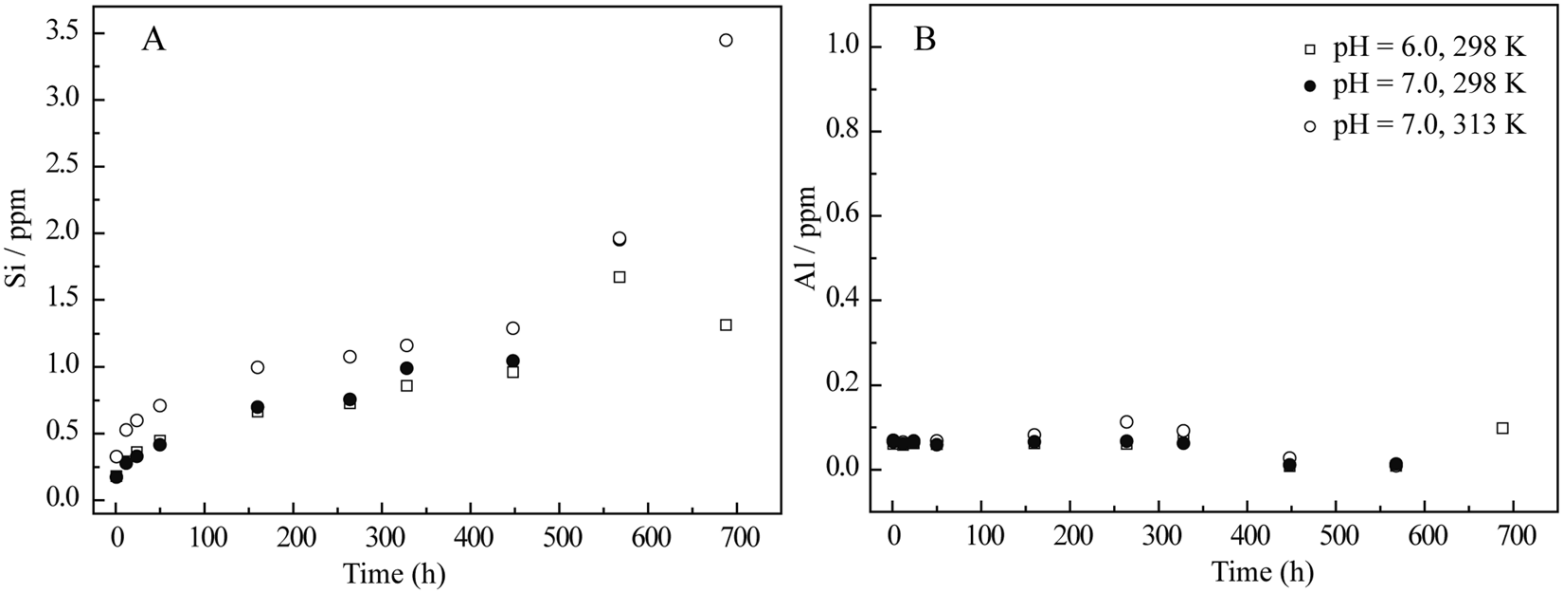
The time-dependence of released Si (ppm) and Al (ppm) in an illite/water system that is Ni(II)-free. *V*_*total*_ = 100 mL, *S/L* = 2.0 g/L, *I* = 0.01 M NaClO_4_.

The structural parameters obtained using EXAFS analysis are also summarized in Table 2. Ni was surrounded by ~6.0 O atoms at *R*_Ni-O_ ≈ 2.05–2.06 Å in the first shell for all the samples, indicating that Ni is in an octahedral environment^25, 30, 33, 34^. For the samples at pH 6.0 with a contact time of 1 day, the second shell was fitted with the Ni-Si scattering pair rather than Ni-Ni and Ni-Al, possibly due to (i) the limited availability of free Al^3+^ within a short contact time (Fig. 6) and (ii) the pH being too low to form any precipitates. Therefore, the Ni-Si scattering pair was considered for the second shell in the RSFs. EXAFS analysis showed that *R*_Ni-Si_ was 3.23 Å with a *CN* of ~3.0 Si atoms, which confirmed the more stable tridentate surface complexes of Ni(II) formed on the illite surface.

For the sample with a contact time over 1 month, the best fit of the second shell includes both the Ni-Ni and Ni-Al scattering pairs. From the *k* ^3^ *χ*(*k*) function, a distinctive beat pattern at ~7.5 Å^−1^ was observed that unequivocally identified the Ni-Al LDH^33, 34^. The central Ni was surrounded by 5.2 Ni atoms with *R*_Ni-Ni_ ≈ 3.08 Å and 0.8 Al atoms with *R*_Ni-Al_ ≈ 3.14 Å. In comparison to the standard Ni-Al LDH sample parameters and the abovementioned features of *k* ^3^ *χ*(*k*) function, the formation of Ni-Al LDH was identified at pH 6.0 with a contact time over 1 month. This phenomenon might be caused by the available Al dissolved by the sorbent substrate. With increasing time, surface precipitates might form on illite and then capture the dissolved Al^3+^ to nucleate a small mass of Ni-Al LDH (Fig. 6).

From the sorption edge (Fig. 2), the amount of Ni(II) uptake increased with increasing pH; thus, striking oscillations were observed in the *k* ^3^χ(*k*) functions and RSFs at pH 7.0. For samples at pH 7.0 with 1 day of contact time, the second shell fitting results showed only Ni-Ni and that the number of second neighbor Ni atoms (*CN*_Ni-Ni_) was 4.0 at *R*_Ni-Ni_ ~ 3.07 Å, which was shorter than the *R*_Ni-Ni_ of β-Ni(OH)_2_ (~3.11 Å) and close to the *R*_Ni-Ni_ of α-Ni(OH)_2_ (3.07–3.09 Å)^27, 35^. This suggested that the Ni precipitate in the early stage was α-Ni(OH)_2_ without available Al^3+^ due to the insufficient Al^3+^ dissolved from the sorbent substrate. After 1 month, Ni was surrounded by 6.0 O atoms (*R*_Ni-O_ ≈ 2.06 Å) in the first shell and 5.2 Ni atoms (*R*_Ni-Ni_ ≈ 3.07 Å) as well as 1.8 Al atoms (*R*_Ni-Al_ ≈ 3.14 Å) in the second shell. This result suggested that Ni-Al LDH was formed as the contact time increased. Scheidegger and Sparks^25^ reported that both adsorption and nucleation processes (mixed Ni/Al phase formation) can occur simultaneously over time scales of only minutes; however, this is suspicious given that the formation process of Ni-Al LDH is related to the kinetics of Al^3+^ dissolution from the illite surface. Unlike the previous studies, we thought that α-Ni(OH)_2_ was formed initially and then progressively converted to the more stable formation of Ni-Al LDH. For the samples at pH 10.0, the contact time had no effect on the second shell in the RSFs, and the *CN*_Ni-Ni_ was 6.0 at *R*_Ni-Ni_ ≈ 3.13 Å, which suggested that β-Ni(OH)_2_ was the primary sorption species. Conversely, the Ni-Al LDH was possibly formed as well at pH 10.0 with an increase in contact time; however, its contribution was too low to be distinguished using EXAFS analysis in this study. Based on the sorption experiment, EXAFS analysis, and SCM, the ISCs and α-Ni(OH)_2_ were primarily responsible for Ni(II) sorption on illite at pH 6.0 and 7.0 with a short contact time, and as the contact time increased from 1 day to 1 month, the sorption species tended to be the more stable form of Ni-Al LDH. However, β-Ni(OH)_2_ gradually became more ubiquitous as pH increased.

### EXAFS analysis on the effects of temperature

**Figure 7:**
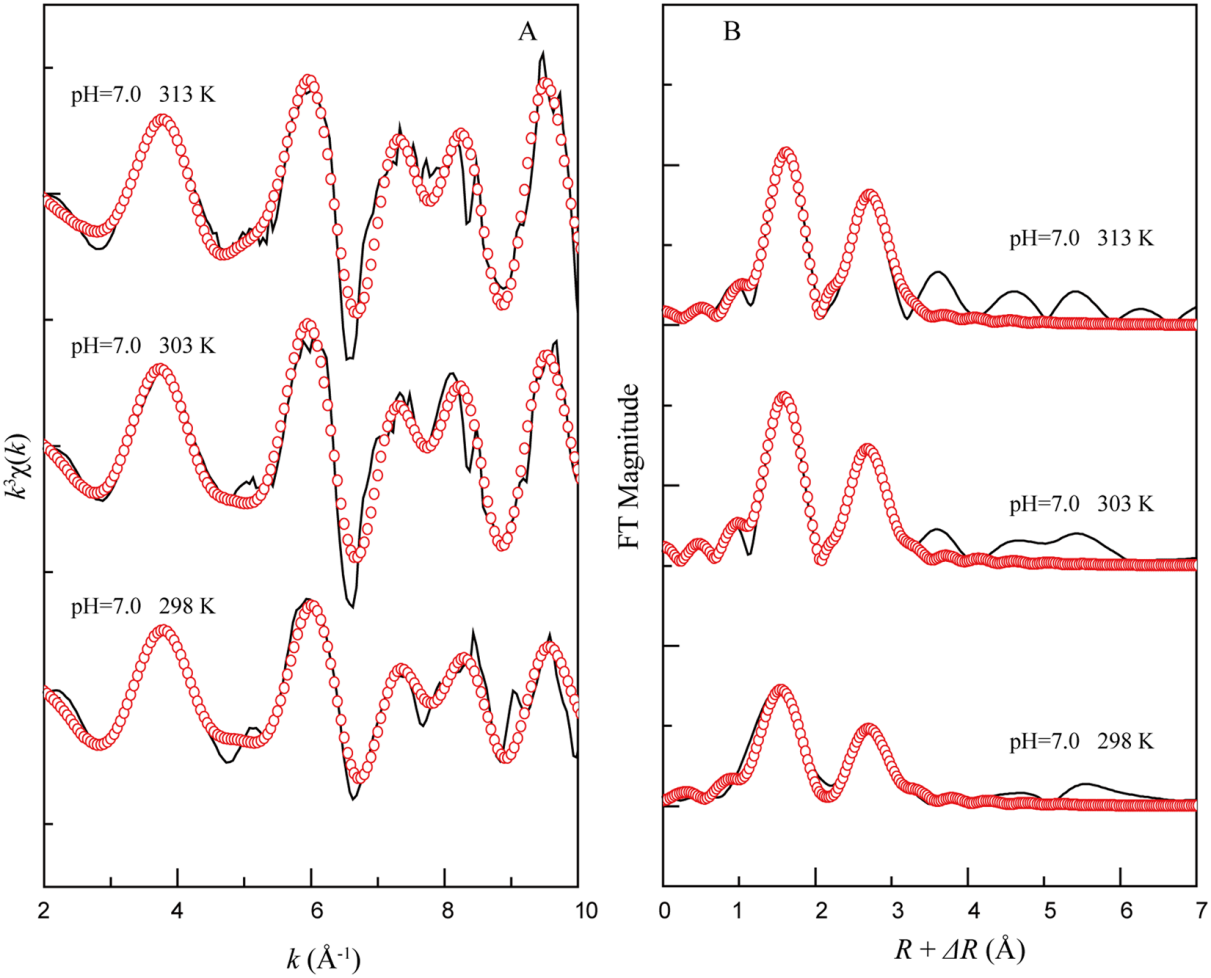
Nickel K-edge EXAFS spectra for Ni(II) adsorbed illite under different temperatures. (**A**) *k* ^3^χ(*k*) functions and (**B**) corresponding RSFs (phase shift uncorrected). Solid line: Experimental data, red dotted line: Fitted data. *C*_Ni(II)initial_ = 1.7 × 10^−3^ mol/L, *pH* = 7.0, *T* = 298 K, 303 K, 313 K, *I* = 0.01 M NaClO_4_, *S/L* = 0.16 g/L.

Figure 7 shows the *k* ^3^χ(*k*) functions and RSFs at different temperatures. The oscillation of the *k* ^3^χ(*k*) function was similar to the Ni precipitate references, and no significant differences were observed at different temperatures (Fig. 7A); however, the corresponding RSFs exhibited an increased intensity for the second shell (Fig. 7B). The structural parameters show that the interatomic distances between Ni and O (*R*_Ni-O_ ≈ 2.05 Å) and *CN*_Ni-O_ were not affected by the increasing temperature, while the coordination environment in the second shell significantly changed with increasing temperature, which indicated that the Ni(II) species changed on the illite surface. At 298 K, the Ni-Ni backscattering pair was dominant, and Ni was surrounded by 4.0 Ni atoms at *R*_Ni-Ni_ ≈ 3.07 Å, indicating the formation of α-Ni(OH)_2_ as discussed above. Moreover, the *CN*_Ni-Al_ at *R*_Ni-Al_ ≈ 3.14 Å increased from 1.5 to 3.2 and the ratio of *CN*_Ni-Ni_/*CN*_Ni-Al_ reduced from 3.7 to 1.8 as the temperature increased from 303 to 313 K, demonstrating the growth of the Ni-Al LD phase. Sheng *et al*.^21^ and Ren *et al*.^33^ also found that Ni(II) surface precipitates were favored at high temperatures. This is reasonable given that the dissolved Al^3+^ has higher availability at high temperatures (Fig. 6), which benefits the formation of Ni-Al LDH; additionally, Ni-Al LDH is a more thermodynamically stable species compared to the other Ni precipitates such as α- or β-Ni(OH)_2_.

### Extraction experiment

**Figure 8:**
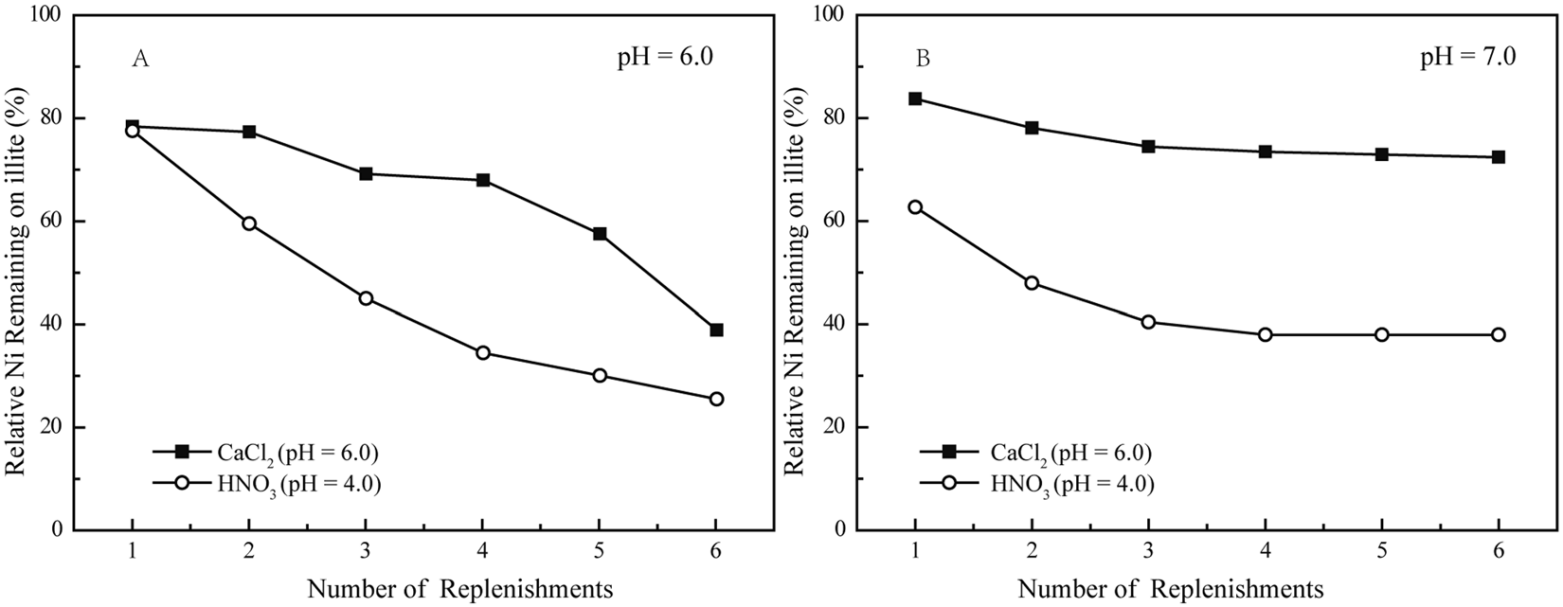
The relative amounts of remaining Ni following the extraction experiments. *C*_Ni(II)initial_ = 1.7 × 10^−3^ mol/L, *S/L* = 2.0 g/L, *T* = 298 K, *I* = 0.1 M NaClO_4_. (**A**) *Cs*(CaCl_2_) = 4.9 × 10^−5^ mol/g, (*Cs* is the initial concentration of Ni(II) on illite before extraction); *Cs*(HNO_3_) = 4.5 × 10^−5^ mol/g, (**B**) *Cs*(CaCl_2_) = 1.8 × 10^−4^ mol/g, *Cs*(HNO_3_) = 1.5 × 10^−4^ mol/g.

Two desorption agents were used: 0.1 M CaCl_2_ (pH = 6.0) and HNO_3_ (pH = 4.0). A CaCl_2_ solution can remove any weakly held ion exchange or surface complexes^40^. An HNO_3_ solution is the most likely to remove Ni proton competition and promote the dissolution of the precipitate phases^31^. Figure 8A shows that both extraction tendencies resulted in a clear and sustained release of Ni(II) because the reversible sorption mechanisms, including the IE and ISCs, were predominant under acidic conditions. Approximately 60% of the Ni(II) was extracted after six treatments with the 0.1 M CaCl_2_ solution, which approximated the contribution of ≡X_2_Ni^0^ to Ni(II) sorption under the observed conditions as shown in Fig. 3. The HNO_3_ solution was significantly more effective for extracting Ni(II) from illite (~70%) compared to the CaCl_2_ solution because both IE and ISCs could be extracted using H^+^. Figure 8B shows that CaCl_2_ released almost 25% of the Ni(II) from illite after six treatments, which was possibly related to the IE, and the remaining 75% of the Ni(II) was possibly related to (co)precipitates (α-Ni(OH)_2_) or the hydrolyzed Ni(II) surface complexes (≡S^w^ONiOH^0^). In the 1.0 M HNO_3_ solution, 63% of the Ni(II) was removed in the six total treatments, indicating the release of IE, ISCs and the partial dissolution of α-Ni(OH)_2_. The remaining phase might be caused by (co)precipitates that have a proton-resistance and cannot be completely dissolved by HNO_3_ (pH = 4.0). These results were consistent with sorption model and EXAFS analyses, suggesting that surface phases and surface complexes were the primary sorption mechanisms at pH 7.0.

### Environmental Implications

**Figure 9:**
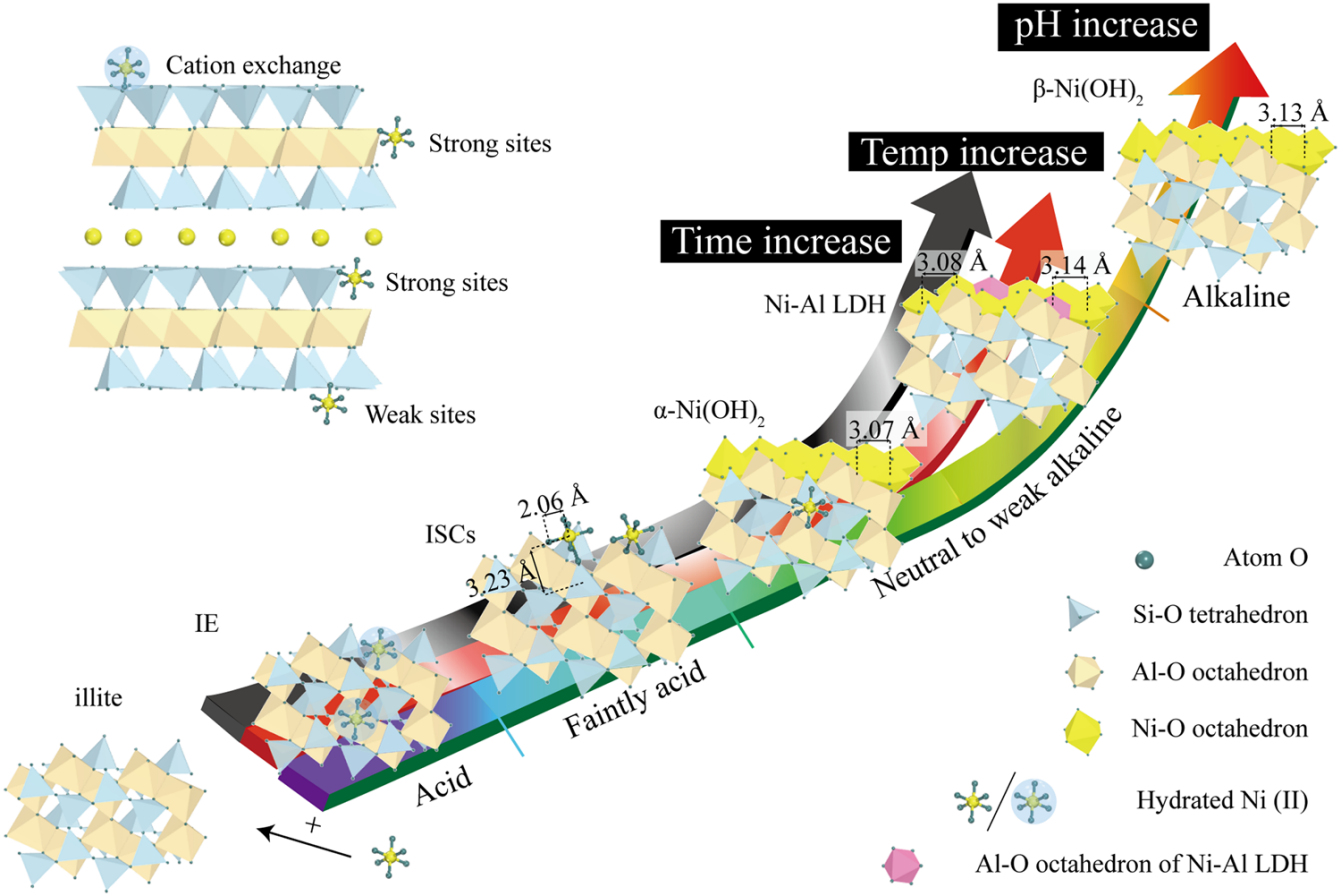
Mechanism and surface site illustrations of Ni(II) sorption on illite. IE, ion exchange; OSCs, outer-sphere complexes; ISCs, inner-sphere complexes; Temp increase, temperature increase.

The sorption mechanisms of heavy metals at the solid/water interface are crucial to their mobility, bioavailability and environmental toxicity. Climatic and environmental conditions typically control heavy metal speciation in the contaminated soil (Fig. 9). For example, the ecologic risk and environmental toxicity of heavy metals in acidic soil will be much higher compared to alkaline soil because the weak heavy metal bonds on soil particles, such as IE or OSCs, are prevalent under acidic conditions as shown in Fig. 9. Similarly, acid rain can also increase the mobility and bioavailability of heavy metals in soil.

The heavy metal content in soil is widespread depending on the surrounding geological environment and anthropogenic and natural activities. At high concentrations, it is well documented that the typical heavy metals such as Zn(II), Co(II), Ni(II), and Fe(II) have similar sorption mechanisms and can readily form mixed metal-Al-layered double hydroxide (LDH) precipitates during sorption with Al-rich soil clays under neutral to weak alkaline conditions^21, 24–34, 41–45^. Kinetic studies on Zn(II)-, Co(II)-, Ni(II), and Fe(II)-Al LDH have shown increased stability of those precipitates on the scale of 1 day. Conversely, these (co)precipitates have special features. For instance, Voegelin and Kretzschmar^42^ reported the formation of Zn-Ni LDH in the simultaneous presence of two metal cations (e.g., Ni and Zn) in soil, and Starcher *et al*.^43^ showed the potential formation of the Fe(II)-Al/Fe(III) LDH phase with Fe(III) impurities in suboxic and anoxic geochemical systems. The abovementioned (co)precipitates significantly decrease metal mobility and bioavailability in contaminated soils.

Moreover, our results confirmed that temperature has an impact on the sorption of heavy metals and the formation of (co)precipitates under neutral to weak alkaline conditions (Fig. 9). Several mentioned studies and this study observed that the initial α-Ni(OH)_2_ might transform into Ni-Al LDH due to the increased loading of Ni(II) and the dissolution of Al^3+^ with an increase in temperature^34^. These findings provide a theoretical basis and scientific guidance for the remediation and purification of heavy metal contaminants in soil; few previous studies considered this point. The precipitation reactions mentioned here may be particularly significant to the speciation and mobility of heavy metals (e.g., Ni, Co, Zn and Fe) in contaminated soil. Additional characteristics and reactions under different conditions, such as the initial concentration, particle size and *S/L* ratio, are important to assess and predict the effects of these precipitate phases in environmental systems.

## Materials and Methods

### Materials

All chemicals used in the experiments were purchased at analytic purity and used directly without any further purification. The illite used in this study was obtained from the Rochester Shale, crushed, and passed through a 200-mesh sieve prior to the experiments. The cation exchange capacity (*CEC*) and N_2_-BET specific surface area are approximately 20.0 meq/100 g and 28.0 m^2^/g, respectively. Additionally, the XRD, FTIR and SEM analyses are shown in the SI.

### Sorption procedure

Ni(II) sorption was performed using batch technology in 10.0 mL polyethylene test tubes, and all stock solutions were prepared in Milli-Q water. A 2.0 g/L slurry was created by mixing illite with deionized water containing 0.1 M NaClO_4_ as a background electrolyte. The stock suspension of illite and NaClO_4_ were pre-equilibrated for 24 h, and the Ni(II) stock solution (*C*_*Ni*_ = 1.7 × 10^−2^ mol/L) was spiked to achieve the desired concentrations; the total volume of each sorption system was maintained at 6.0 mL. The pH was adjusted to the desired value by adding a negligible HClO_4_ or NaOH solution. After the suspensions were shaken for 24 hours, 1 week, 1 month, or 3 months, the solid and liquid phases were separated by centrifugation at 12,000 rpm for 30 min. The concentration of Ni(II) in the supernatant (*C*_e_) was analyzed via spectrophotometry at 530 nm using a Ni butanedione dioxime complex.

### Potentiometric titration of illite

Previous studies have confirmed three types of sorption sites on illite surfaces, including a strong sorption site (≡S^s^OH) that has a very low density and high reactivity, a weak sorption site (≡S^w^OH) that has a high density and low reactivity, and an IE site (≡XNa)^37–39, 46^. In this study, the site capacity and intrinsic complex constants (*K*) of the strong and weak sites were calculated by fitting the potentiometric titration data (Fig. SI-3) using MINTEQ 3.1 code. The classical constant capacitance model (CCM) was generally applied to describe the surface amphoteric sites (i.e., surface hydroxyl) and the fixed-charge site was used to describe the IE. The capacity of the weak site (≡S^w^OH) was 1.0 × 10^−1^ mol/kg and the strong site (≡S^s^OH) had a capacity that was approximately 4.0% of the weak site. The capacity of the IE site (≡XNa) listed in the fixed-charge site was 2.0 × 10^−1^ mol/kg and used the gt_std.cdb database combined with the MINTEQ 3.1 code. The fit results showed that the titration curve can be adequately simulated and the relative parameters are comparable to previous studies^37–39^, which will be used as constants in the following modelling for Ni(II) sorption edges on illite.

### EXAFS measurement

The procedures for the EXAFS samples and spectra were performed following the method of Qiang *et al*.^47^. The detailed methods are shown in the SI-3. Nickel K-edge EXAFS spectra at 8333.0 eV were collected at the BL14W1 station of the Shanghai Synchrotron Radiation Facility (SSRF, Shanghai, China) and the BL12C station of the KEK Photon Factory (Tsukuba, Japan). The EXAFS spectra of Ni(NO_3_)_2_, β-Ni(OH)_2_, Ni-phyllosilicate and Ni-Al LDH were collected under the transmission mode, whereas the Ni(II)-adsorbed samples were measured under the fluorescence mode using a multi-element, high-purity Ge solid-state detector (32-element SSD for the BL14W1 at the SSRF and 19-element SSD for the BL12C at the KEK-PF). The normalization of the EXAFS spectra and data analysis were reduced using standard procedures^48^ and were performed with the aid of the Athena and Artemis interfaces compacted in the IFEFFIT^49^ and FEFF 7.0^50^. During the optimization, the energy shift (Δ*E*_0_) was constrained to be equal, and the amplitude reduction factor, (*S*_0_^2^), was fixed at 0.85. A good fit was determined based on the minimum residual factor (*R*_f_)^40^.

### Extraction procedure

Extraction experiments were performed using the modified sequential extraction following the procedures by Nachtegaal *et al*.^40^, Peltier *et al*.^31^ and Fan *et al*.^51^. In general, two types of extraction solutions were chosen in this study: 0.1 M CaCl_2_ (pH = 6.0) (IE fraction) and HNO_3_ (pH = 4.0) (proton-promoted dissolution). Two Ni(II)-adsorbed samples were prepared at pH 6.0 and 7.0, which were strictly identical to those used in the adsorption experiments in Section 2.2. After the sorption experiment, the solid was rinsed rapidly using Milli-Q water for the subsequent extraction experiments. For each extraction experiment, 15.0 mL of the extraction agent was added to the polyethylene test tubes and continuously shaken. For the first treatment, a contact time of 12 hours was used to minimize potential resorption of Ni to the solid phases. Then, all subsequent treatments used a 24-hour contact time. Afterwards, the batches were centrifuged, and the supernatant was collected for Ni analysis. The remaining solids were then washed with Milli-Q water and centrifuged prior to the addition of an extraction solution.

## Electronic supplementary material

Supplementary Information
