# Supplementary material for: Exploring the Sorption Mechanism of Ni(II) on Illite: Batch Sorption, Modelling, EXAFS and Extraction Investigations
Source: Sci Rep. 2017 Aug 17;7:8495. doi: 10.1038/s41598-017-09188-z (PMC5561197; doi:10.1038/s41598-017-09188-z)
Supplement: Supplementary file 1 — Supplementary Information [file 41598_2017_9188_MOESM1_ESM.pdf]

Supplementary Information on

Exploring the Sorption Mechanism of Ni(II) on Illite: Batch Sorption, Modelling, EXAFS and Extraction Investigations

Xiaolan Zhao[1,5], Shirong Qiang[2], Hanyu Wu[1], Yunbo Yang[1,5], Dadong Shao[3], Linchuan Fang[4], Jianjun Liang[1], Ping Li[1], Qiaohui Fan[1,*]

[1] *Key Laboratory of Petroleum Resources, Gansu Province / CAS Key Laboratory of Petroleum Resources Research, Institute of Geology and Geophysics, Chinese Academy of Sciences, Lanzhou, Gansu, 730000, China;*

[2] *Key Laboratory of Preclinical Study for New Drugs of Gansu Province, and Institute of Physiology, School of Basic Medical Sciences, Lanzhou University, 199 Donggang West Road, Lanzhou 73000, China;*

[3] *Institute of Plasma Physics, Chinese Academy of Sciences, Hefei 230031, China;*

[4] *State Key Laboratory of Soil Erosion and Dryland Farming on the Loess Plateau, Northwest A&F University, Yangling 712100, China;*

[5] *Graduate University of Chinese Academy of Sciences, Beijing, 100049, China.*

* Corresponding author. Tel: +86-931-4960-831. E-mail: fanqh@lzb.ac.cn or fanqiaohui@gmail.com;

Xiaolan Zhao: zhao_xl08@163.com
Shirong Qiang: qiangshirong@lzu.edu.cn
Hanyu Wu: wuhy13@lzu.edu.cn
Yunbo Yang: 1157565244@qq.com
Dadong Shao: shaodadong@126.com
Lingchuan Fang: flinc629@hotmail.com
Jianjun Liang: liangjj@lzb.ac.cn
Ping Li: lipingls06@126.com

SI-1. Characterization of illite

Illite was achieved from the Rochester Shale in this study. Imt-1 was one of the common illite achieved from Cambrian shale (Silver Hill, Montana) as a reference. As can be seen from Figure SI-1 that the characteristics of XRD and FTIR, illite and Imt-1 had similar characteristics in structure and topology.

.

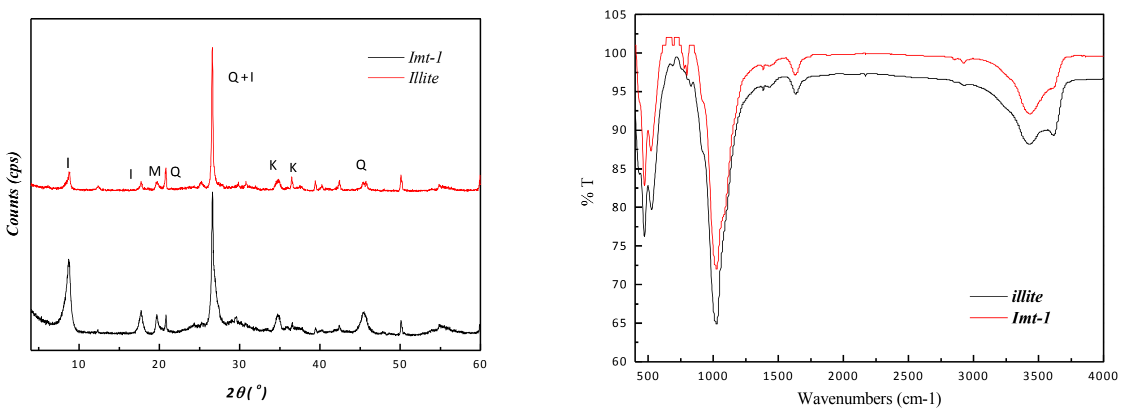

Figure SI-1. XRD and FTIR patterns of illite and Imt-1.

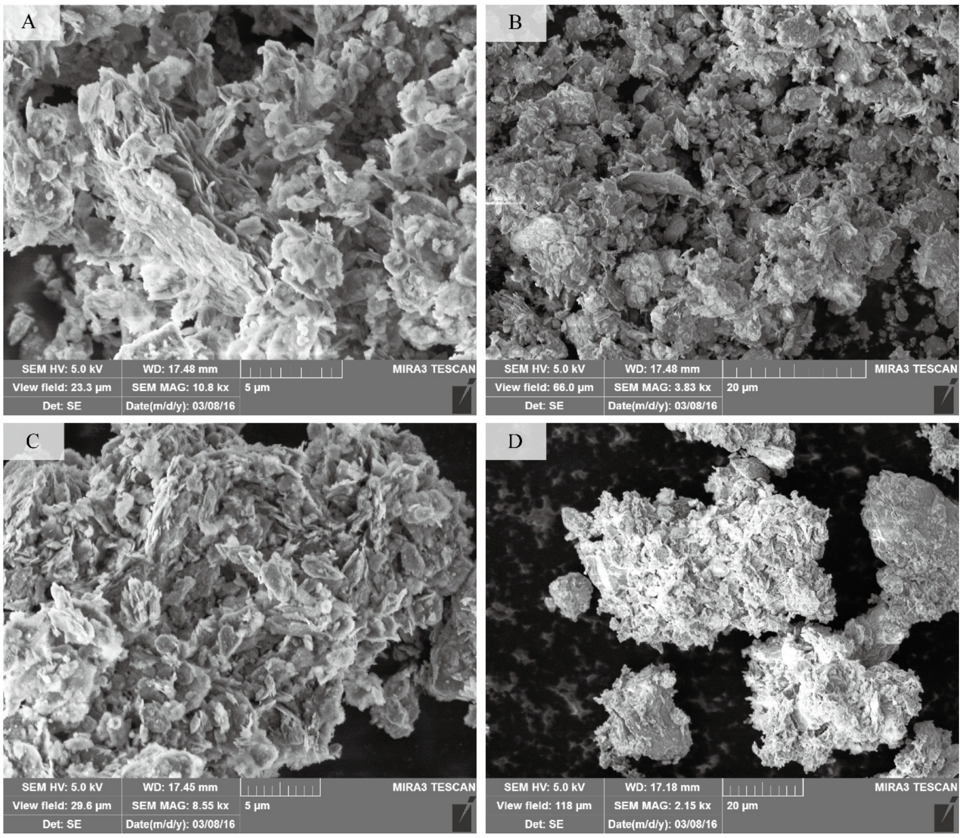

Figure SI-2. SEM images of illite. A, pure illite (5 μm); B, pure illite (20 μm); C, Ni(II) sorption on illite at pH 6.0 (5 μm); D, Ni(II) sorption on illite at pH 10.0 (20 μm).

SI-2. Acid–base titration of of illite

Potentiometric titration of aqueous illite suspension (2.0 g/L) in 0.01 mol/L $NaClO_4$ solution were carried out under argon atmosphere and at 20 °C with a Mettler-Toledo DL50 titrator equipped with a combine electrode (glass electrode associated to a reference electrode Ag/AgCl/KCl 3.0 mol/L). The electrode was calibrated in pH 4.00, pH 6.86 and pH 9.18 buffers (Alfa Aesar, specpure). Before the titrations were started, the suspensions were titrated up to about pH 3.0 with 0.0736 mol/ L HNO_3 solution and purged with argon gas for about 1 h. Then the titrations were started from pH about 3.0 to pH 10.5 with 0.0482 mol/L NaOH solution. The total concentration of consumed proton (TOTH) in the titration process was calculated by Gran-plot functions, which has been described well in our previous work[1].

In this study, the classical constant capacitance model (CCM) combing one kind of fixed-charged site was used to describe the surfaces of hydroxyl. The capacity of weak site ($\equiv S^{w}OH$) was 1.0×10^{-1} mol/kg and the strong site ($\equiv S^{s}OH$) have a capacity approximately 4.0% that of the weak site. Capacity of IE site (≡XNa) listed in the fixed-charged site was 2.0×10^{-1} mol/kg and used the gt_std.cdb database combined in MINTEQ 3.1 code. All parameters were not only referred to the previous studies[2-4] but also changed slightly combining with our experimental data, which were summarized in Table 1 and will be used as constants in the following modelling for Ni(II) sorption edges on illite.

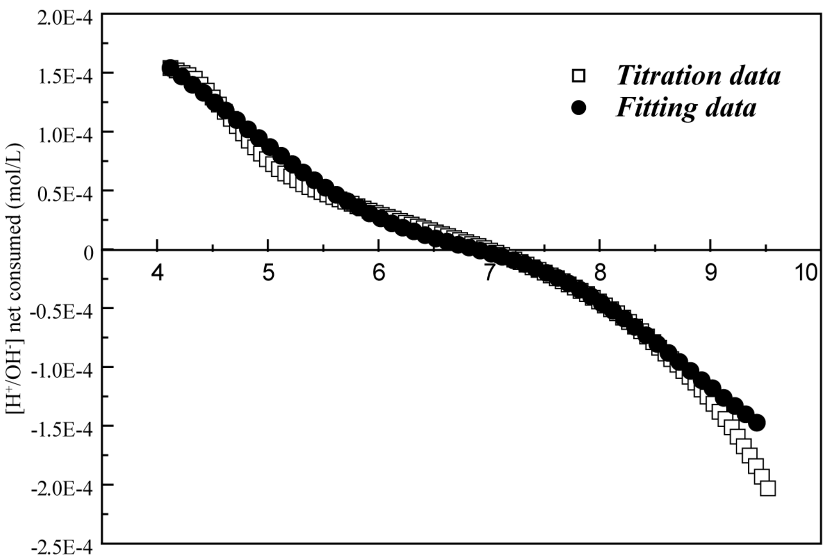

Figure SI-3. Titration data for illite in a 0.01M $NaClO_4$ solution.

SI-3. EXAFS samples preparation, data collection and analysis

(a) Preparation of EXAFS samples. Illite (0.08 g) was saturated with a 1.7×10^{-3} mol/L Ni(II) solution at different pH (6.0, 7.0 and 10.0) for 1 day or 1 month. After the Ni sorption reached equilibrium, the final suspension was separated using the previously mentioned procedure. The solid phase was carefully rinsed with Milli-Q water twice to remove the residual Ni(II) solution and then sealed into polyethylene bags for the EXAFS measurement. The supernatant was used to determine the Ni(II) concentration.

(b) EXAFS measurement. Nickel K-edge EXAFS spectra were collected at the BL14W1 station of the Shanghai Synchrotron Radiation Facility (SSRF, Shanghai, China) and the BL12C station of the KEK Photon Factory (Tsukuba, Japan). For the BL14W1 station in the SSRF, the electron beam energy was 3.5 GeV, and the mean stored current was 220 mA. The X-ray beam was diffracted by a double-crystal Si (111) monochromator that passes a narrow energy band to the samples. Two Rh-coated mirrors placed downstream of the monochromator were used to detune the high-order harmonics. Ionization chambers with N_2 atmosphere were used to collect the nickel K-edge spectra in transmission mode at room temperature. In the case of the BL12C station in the KEK-PF, the electron beam energy was 2.5 GeV with a

maximum current of 450 mA. The X-ray energy was tuned using a fixed-exit double-crystal Si (111) monochromator. Before X-ray absorption spectrum (XAS) collection, the absorption edge was calibrated by assigning the first inflection point on the K-edge of a nickel metal foil to 8333.0 eV. The XAS data of $Ni(NO_3)_2$, $Ni(OH)_2$, and Ni-Al LDH were collected under transmission mode, whereas the Ni(II)-adsorbed samples were measured under fluorescence mode using a multi-element, high-purity Ge solid-state detector (32-element SSD for the BL14W1 at the SSRF and 19-element SSD for the BL12C at the KEK-PF). Moreover, all spectra were collected at room temperature, and at least three scans were collected per sample to improve the signal-to-noise ratio[5].

(c) EXAFS analysis. The EXAFS spectra were reduced using standard procedures[6] and were performed with the aid of the Athena and Artemis interfaces compacted in the IFEFFIT[7] and FEFF 7.0[8]. Fourier transforms (FTs) were obtained from the $k^3\chi(k)$ functions after apodization by a Kaiser-Bessel window, and then the radial structure functions (RSFs) were achieved within a *k*-range of 1.5-12.0 Å^{-1}. Two major shells below 3.5 Å in the RSFs were isolated and Fourier back-transformed for spectral simulation. Structural parameters were extracted with fits to the standard EXAFS equation. Ab initio Ni-O and Ni-Ni/Al scattering paths were generated using the FEFF 7.0 from the refinement of the structure of lizardite where Ni was substituted for Mg in octahedral positions. During the optimization, the energy shift (ΔE_0) was constrained to be equal, and the amplitude reduction factor, (S_0^2), was fixed at 0.85. A good fit was determined based on the minimum residual factor (R_f)[5].

SI-4. Effect of solid content

The sorption of Ni(II) on illite as a function of solid content was shown in Fig. SI-4. It can be seen that the sorption percentage of Ni(II) was increased with the increasing of illite content. This was consistent with previous studies[9, 10]. With increasing solid content, the functional groups at illite surfaces increased and thereby provided more sites to cooperate with metal ions.

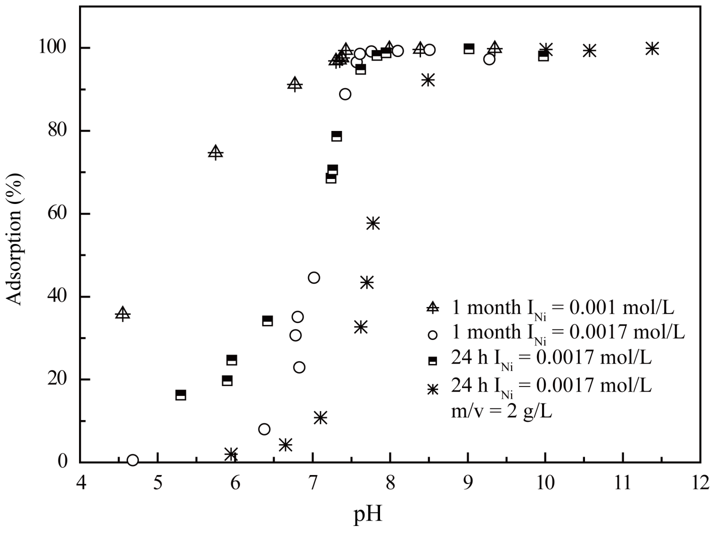

Figure SI-4. Sorption of Ni(II) on illite as a function of solid content. $C(\mathrm{Ni})_{\mathrm{initial}}$ = 1.7×10^{-3} mol/L, T = 298 K, I = 0.1 M $NaClO_4$, S/L = 10.0 g/L.

SI-5 Sorption isotherms

The sorption isotherms of Ni(II) on illite at 303 and 313 K were shown in Fig. SI-5. One can see that the uptake of Ni(II) increased with rise in temperature, indicating that high temperature was favorable for Ni(II) sorption on illite. The sorption isotherms of Ni(II) at 303 and 313K were correlated by the Langmuir and Freundlich models, respectively, and related parameters were shown in Table SI-1. The Langmuir model, describing homogeneous sorption, fitted the sorption process better than the Freundlich model at low temperature. At high temperature, Freundlich is better, indicating that changes in the mechanism of Ni(II) sorption on illite were expected in the higher temperature.

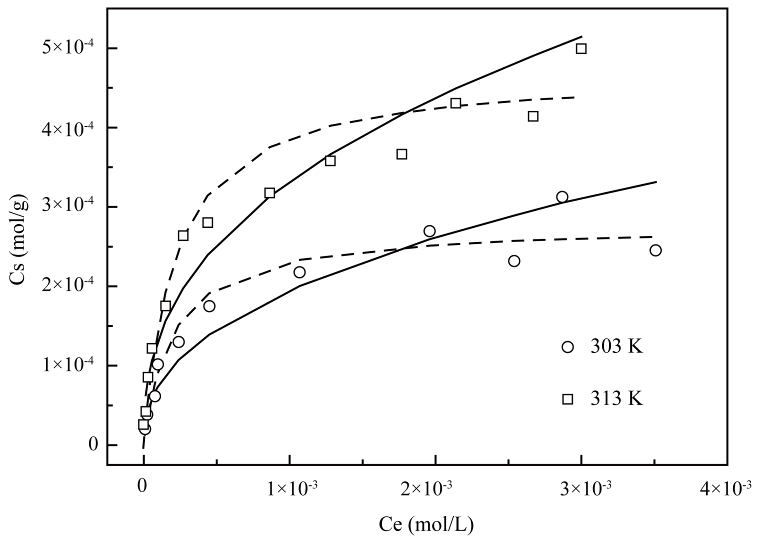

Figure SI-5. Sorption isotherms at different temperatures. $pH = 7.0$, $I = 0.01$ M $NaClO_4$, $S/L = 2.0$ g/L. Solid line: Freundlich fitting, dash line: Langmuir fitting.

Table SI-1

Relative Parameters of Langmuir and Freundlich Models of Ni(II) sorption on illite

models	parameters	Temperature	
		303 K	313 K
Langmuir model (Langmuir, 1918)	q_{max} (mol/g)	2.8×10^{-4}	4.7×10^{-4}
$\frac{C_e}{q_e} = \frac{1}{K_L q_{max}} + \frac{C_e}{q_{max}}$	K_L (L/g)	4.91×10^{3}	4.59×10^{3}
	R^2	0.9727	0.972
Freundlich model (Freundlich, 1906)	K_F ($mol^{1-n}L^n/g$)	3.6×10^{-3}	5.2×10^{-3}
$\log q_e = \log K_F + n \log C_e$	n	0.422	0.400
	R^2	0.9436	0.9446

SI-6 XPS measurement and analysis

The XPS measurements were performed on a PerkinElmer PHI-5702 multifunctional X-ray photoelectron spectroscope, using an Al Kα monochromatized source (hv = 1253.5 eV) and a multidetection analyzer under a 10^{-6} Pa residual pressure. XPSPEAK 4.1 was used to analyze the spectra. Surface charging effects were corrected with the C 1s peak at 284.6 eV as a reference. Shirley background correction and Gaussian-Lorentzian fitting were used to transform peak areas to total intensities.

Different pH values resulted in different energies/peaks in the XPS spectra, which are shown in Fig. SI-6. As seen from Fig. SI-6B, the Ni 2p spectrum exhibited two contributions, $2p_{3/2}$ and $2p_{1/2}$, which had peak shifts with the increased pH values. Ni 2p at pH ~6.0 appeared as Ni $2p_{3/2}$ at 856.9 eV and Ni $2p_{1/2}$ at 874.5 eV, while 2p at pH ~7.0 appeared as Ni $2p_{3/2}$ at 856.8 eV and Ni $2p_{1/2}$ at 874.3 eV. Such 0.1 and 0.3 eV peak shifts might be caused by the changes of sorption mechanism. Combining the modelling and EXAFS analysis, the ISC of $\equiv S^{w}ONi^{+}$ and IE of $\equiv X_2Ni^{0}$ were dominant at pH ~6.0, and surface precipitates occurred at pH ~7.0. The XPS results were consistent with the modelling and EXAFS analysis. At pH ~10.0, the binding energy for Ni $2p_{3/2}$ reduced to 855.80 eV and Ni $2p_{1/2}$ reduced to 873.4 eV, indicating the features of $Ni(OH)_2$[11]. From Fig. SI-6A, it is worth noting that the intensity of Al 2p increased clearly at pH ~10.0, suggesting that the Al^{3+} dissolution increased. This meant that there may have been Ni-Al LDH at pH ~10.0, whose features in the EXAFS spectra were covered up by a mass of β-$Ni(OH)_2$. Fig. SI-6C shows that the shift in binding energies provided solid evidence of the change in sorption mechanism at different pH values. Compared with the sample at pH ~6.0, the curve of the sample at pH ~7.0 presented an inflection point at ~530.5 eV, the same as the sample at pH ~10.0, demonstrating the contribution of (co)precipitates. Three samples had four O 1s peaks positioned at 530.98 – 530.7 eV, 531.5 eV, 532.25 eV and 533.1 eV, which can be assigned to lattice oxygen O^{2-}, Al-OH, Si-OH, and adsorbed H_2O. There was no significant difference of binding energy among Al-OH, Si-OH and adsorbed H_2O because of saturated sorption sites. The binding energy of the peaks for lattice oxygen O^{2-} decreased from 530.98 eV to 530.7 eV, indicating that the sorption of Ni(II) can be greatly attributed to the (co)precipitates.

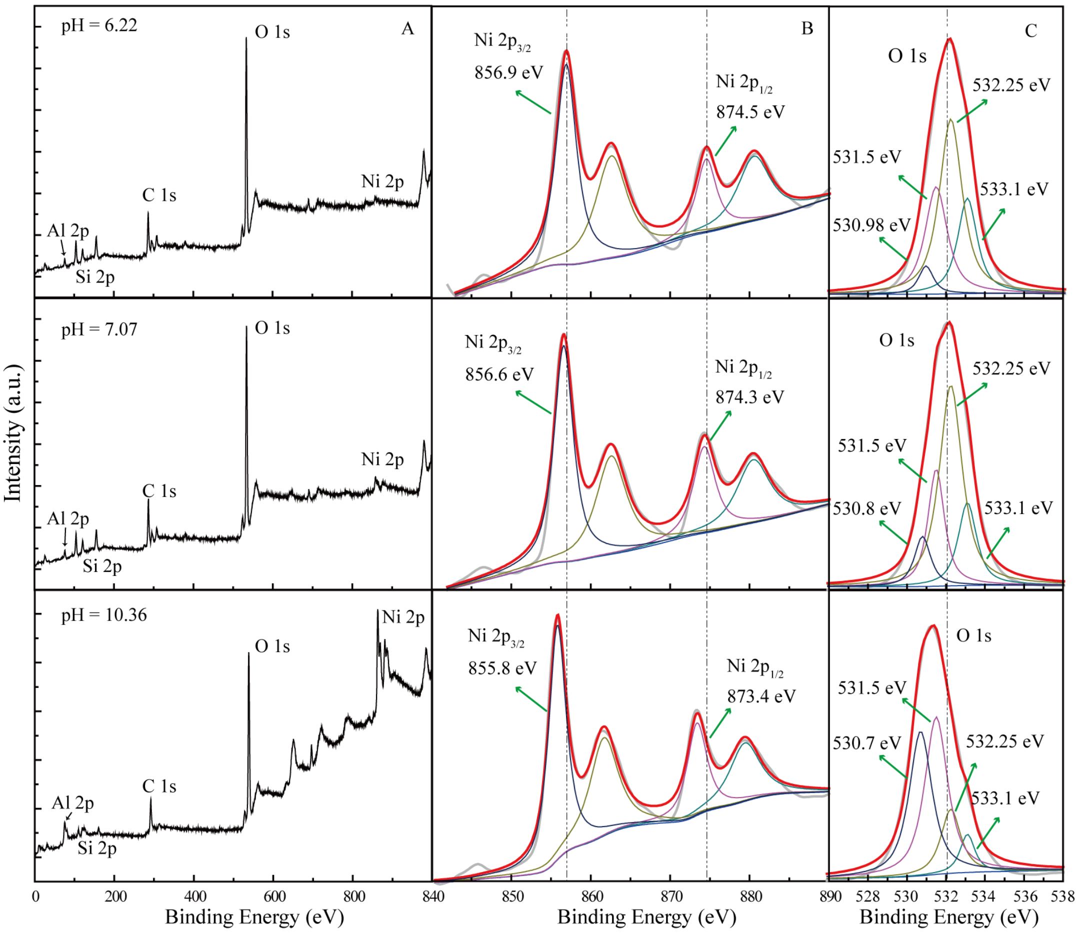

Figure SI-6. XPS spectra of the survey, Ni 2p and O 1s of Ni(II) sorption samples at (A) *pH* = 6.22, (B) *pH* = 7.07, (C) *pH* = 10.36, $C_{Ni(II)initial} = 1.7 \times 10^{-3}$ mol/L, *S/L*= 2.0 g/L, *I* = 0.01 M $NaClO_4$, *T* = 298 K.

References

1 Niu, Z. W. et al. Effect of pH, ionic strength and humic acid on the sorption of uranium(VI) to attapulgite. *Appl. Radiat. Isotopes* **67**, 1582-1590 (2009).

2 Bradbury, M. H. & Baeyens, B. Sorption modelling on illite Part I: Titration measurements and the sorption of Ni, Co, Eu and Sn. *Geochim. Cosmochim.Acta* **73**, 990-10dd03 (2009).

3 Benedicto, A., Degueldre, C. & Missana, T. Gallium sorption on montmorillonite and illite colloids: Experimental study and modelling by ionic exchange and surface complexation. *Appl. Geochem.* **40**, 43-50 (2014).

4 Marsac, R. et al. Neptunium redox speciation at the illite surface. *Geochim. Cosmochim.Acta* **152**, 39-51 (2015).

5 Nachtegaal, M. & Sparks, D. L. Nickel sequestration in a kaolinite-humic acid complex. *Environ. Sci. Technol.* **37**, 529-534 (2003).

6 Teo, B. K. EXAFS: Basic Principles and Data Analysis. *Springer-Verlag, Berlin* (1986).

7 Ravel, B. & Newville, M. ATHEAN, ARTERMIS, HEPHAESTUS; data analysis for X-ray

absorption spectroscopy using IFEFFIT. *J. Synchrotron. Radiat.* **12**, 537-541 (2005).

8 Zabinsky, S. I., Rehr, J. J., & Ankudinov, A. Multiple-scattering calculations of X-ray-absorption spectra. *Phys. Rev. B* **52**, 2995-3009 (1995).

9 Fan, Q. H., Shao, D. D., Lu, Y., Wu, W. S. & Wang, X. K. Effect of pH, ionic strength, temperature and humic substances on the sorption of Ni(II) to Na-attapulgite. *Chem. Eng. J.* **150**, 188-195 (2009).

10 Hu, B. W., Cheng, W., Zhang, H. & Yang, S. T. Solution chemistry effects on sorption behavior of radionuclide Ni-63(II) in illite-water suspensions. *J. Nucl. Mat.* **406**, 263-270 (2010).

11 Ali-Loytty, H. et al. Ambient-Pressure XPS Study of a Ni-Fe Electrocatalyst for the Oxygen Evolution Reaction. *J. Phys. Chem. C* **120**, 2247-2253 (2016).
